# Effects of cardiac output levels on the measurement of transpulmonary thermodilution cardiac output in patients with acute lung injury

**DOI:** 10.1186/cc10824

**Published:** 2012-03-20

**Authors:** K Kao, H Hu, C Hung, C Chang, C Huang

**Affiliations:** 1 Chang Gung Memorial Hospital, Kwei-Shan,Taoyuan, Taiwan

## Introduction

Transpulmonary thermodilution cardiac output (CO) correlates closely with pulmonary artery (PA) thermodilution CO. Levels of CO may contribute varying amounts of thermal indicator loss and recirculation during thermodilution CO measurement. This study aimed to investigate the effects of CO levels on the agreement between transpulmonary and PA thermodilution CO in acute lung injury (ALI) patients.

## Methods

Twenty-two ALI patients were prospectively enrolled. Paired bolus transpulmonary thermodilution cardiac index (BCItp) and continuous PA thermodilution cardiac index (CCIpa) data were recorded at baseline and repeated immediately and at 2, 4, and 6 hours after volume expansion with a 500 ml infusion of 10% pentastarch (HES 200/0.5).

## Results

One hundred and ten paired CI measurements were recorded and divided into four quartiles from the lowest to the highest CCIpa. The mean BCItp was higher than CCIpa, and the Bland-Altman analysis revealed a bias of 0.57 ± 0.75 l/minute/m^2^. The limits of agreement (2SD) were +2.07 to -0.93 l/minute/m^2^. BCItp correlated closely with CCIpa (*R *= 0.887). CCIpa negatively correlated with the difference between BCItp and CCIpa (*R *= -0.26). The bias of quartile 1 with the least CCIpa was significantly greater than those of the three other quartiles.

## Conclusion

In ALI patients, transpulmonary thermodilution is a clinically acceptable and interchangeable alternative to PA thermodilution for CO measurement. Levels of CO weakly and negatively correlate with the difference between BCItp and CCIpa. There is greater overestimation of BCItp over CCIpa in low than in high CO states.

## References

[B1] Harvey S Lancet 2005 366 472 477 10.1016/S0140-6736(05)67061-4 16084255

[B2] Koo KKY Crit Care Med 2011 39 1613 1618 10.1097/CCM.0b013e318218a045 21494107

